# Century-Old DNA Barcodes Reveal Phylogenetic Placement of the Extinct Jamaican Sunset Moth, *Urania sloanus* Cramer (Lepidoptera: Uraniidae)

**DOI:** 10.1371/journal.pone.0164405

**Published:** 2016-10-20

**Authors:** Vazrick Nazari, B. Christian Schmidt, Sean Prosser, Paul D. N. Hebert

**Affiliations:** 1 Canadian National Collection of Insects, Arachnids and Nematodes, Ottawa Research and Development Centre, Agriculture and Agri-Food Canada, Ottawa, Ontario, Canada; 2 Centre for Biodiversity Genomics, Biodiversity Institute of Ontario, University of Guelph, Guelph, Ontario, Canada; Tel Aviv University, ISRAEL

## Abstract

Analysis of the DNA barcode region of the cytochrome *c* oxidase 1 gene from a specimen of the extinct Jamaican sunset moth, *Urania sloanus*, places this species as a sister to the Central American *U*. *fulgens*. We found that all *Urania* F. species were closely related (<2.8% maximum divergence at COI), with the Cuban endemic *U*. *boisduvalii* appearing as sister to the rest. The low divergence in DNA barcodes and genitalic structures indicate that the Cuban *U*. *poeyi* and eastern Brazilian *U*. *brasiliensis* are geographic segregates of *U*. *fulgens* and *U*. *leilus* respectively, so the former two taxa are accordingly recognized as subspecies.

## Introduction

The Jamaican Sunset moth, *Urania sloanus* (Cramer, 1776), often regarded as one of the most beautiful of all moths, became extinct just 116 years after its description [1,2]. Endemic to Jamaica, it inhabited low-elevation tropical rainforest containing its host plant *Omphalea* L. (Euphorbiaceae), especially *O*. *triandra* L. (Jamaican Cobnut) and *O*. *diandra* L. [3–5]. *Urania sloanus* was named by Dutch entomologist Pieter Cramer [6] in honor of Sir Hans Sloane (1660–1753), an English naturalist and collector who explored Jamaica from 1687 to 1689, recording and illustrating the species for the first time in his travel diaries published in 1725 [7]. The life history and behaviour of *U*. *sloanus* was subsequently described in detail by Philip Gosse [3,8–9], who reported that adults were common from mid-March to late July but usually rare in the winter. Periodic episodes of abundance, when swarms of these moths occurred at flowering trees in the Blue Mountains of Portland, were intervened by years of great scarcity [1,8,10–11]. The moth became increasingly rare in the early 1890s; the last sighting occurred in 1894–1895 [1], although the species may have survived until 1908 [12]. Lees and Smith [11] postulated that populations of *U*. *sloanus* declined below a sustainable level due to the loss of its main larval host through habitat destruction linked to agriculture or hurricanes. Vinciguerra [12] suggested the species may still persist in remote areas with appropriate habitat and host plants.

In addition to *U*. *sloanus*, five other species of *Urania* Fabricius are generally recognized: *U*. *leilus* (Linnaeus, 1758), widespread in tropical South America east of the Andes; *U*. *fulgens* Walker, 1854, a migratory species distributed from Mexico through Central America to northwestern South America (occasionally straying to Florida and Texas [13] as well as Jamaica [Lees, pers. comm.]), and *U*. *boisduvalii* Guérin-Meneville, 1829, endemic to Cuba. The status of two other species, namely *U*. *brasiliensis* (Swainson, 1833) (Atlantic coast of Brazil) and *U*. *poeyi* (Herrich-Schäffer, 1866) (Cuba) is controversial as they have been treated either as separate species [11,14–17], or subspecies or synonyms of *U*. *fulgens* and *U*. *leilus*, respectively [18]. *Urania fulgens* can exhibit forms that are externally very similar to *U*. *leilus*, and these two taxa have also been suggested to be conspecific [19]. By contrast, only *U*. *sloanus* shows pink and red coloration on the dorsal hindwing. The diagnostic value of genitalic structures has not been evaluated in *Urania*.

In addition to the Neotropical genus *Urania*, the pantropical subfamily Uraniinae contains six other genera (*Alcides* Hübner, *Chrysiridia* Hübner, *Cyphura* Warren, *Lyssa* Hübner, *Urapteritra* Viette and *Urapteroides* Moore) with many large, tailed diurnal species with a spectacular phenotype. The family Uraniidae apparently originated in the late Cretaceous, ~75 Mya [20] from a common ancestor with the Sematuridae [21–22] although no comprehensive phylogeny is available for it. Lees and Smith [11] provided a preliminary phylogeny for the Uraniinae based on seven larval and adult tympanic characters, while molecular studies [20–22] have incorporated a few representative uraniid genera within broader lepidopteran phylogenies. *Urania* and *Chrysiridia* were resolved as sister genera by Lees and Smith [11] based on shared larval and tympanic morphology and further supported by the diurnality of their adults, wing scale iridescence, and their shared use of *Omphalea* as larval hosts. A combined molecular and morphological analysis [22], however, found weak support for *Alcides* as the sister group to *Urania*, with both sisters to *Chrysiridia*.

*Urania* moths are unique among Lepidoptera in having stridulatory organs on their male prothoracic legs [23–25]. They are also among the very few moths that form migrant populations, being chemically defended against predators by polyhydroxy alkaloids sequestered from their host plants, *Omphalea* [26–27]. Species in the same genus are also hosts for Afrotropical *Chrysiridia*, as well as the Indo-Australian *Alcides* and *Lyssa* [11,28]. In fact, the disjunct distributions of these moths almost exactly correspond to the global range of *Omphalea*, suggesting a deep historical relationship, perhaps even predating the final break-up of Gondwanaland in the Cretaceous, ca. 100–65 Mya [11].

All *Urania* species are allo- or parapatric with respect to each other and show island endemism (*U*. *boisduvalii*, *U*. *poeyi*, *U*. *sloanus*), but species relationships have never been studied. Here we investigate the pattern of sequence divergence at COI among species of *Urania*, including the extinct *U*. *sloanus*, in the large context of family Uraniidae.

## Methods and Materials

### Samples

40 specimens from all six recognized species of *Urania*, including one specimen of *U*. *sloanus*, were sequenced for COI. In addition, to aid phylogenetic inferences we included 14 representatives from the other six genera of Uraniinae (7), subfamilies Auzeinae (2), Epipleminae (2) and Microniinae (2), as well as family Sematuridae (1), recognized as sister to Uraniidae [21]. Sequences were downloaded from GenBank or BOLD with permission (S1 Table). Genitalia were prepared following the methods of Lafontaine [29]. Male genitalia of all *Urania* taxa except *U*. *sloanus* were examined (n = 5); females were not available for all species. Cleaned, stained genitalia were stored and examined in 30% ethanol, and slide-mounted in Euparal before being photographed using a Leica DFC450 digital camera and the Leica Application Suite 4.7.1 software package.

### Amplification and sequence analysis

A single leg was removed from each specimen and sent to Biodiversity Institute of Ontario in Guelph, Canada for DNA extraction, amplification, and sequencing. Non-destructive DNA extraction was used for the leg from *U*. *sloanus*. All work was performed in a dedicated clean lab using UV sterilized equipment to minimize the risk of contamination. DNA extraction and purification was performed following the methods of Prosser *et al* [30] and the 658 bp COI barcode region was amplified for both Sanger-based sequencing and next-generation sequencing (NGS). For Sanger sequencing, non-tailed primers (S2 Table) were used to amplify six short, overlapping fragments using a modified version of the method described in Lees *et al* [31]. Each of the six fragments from each sample were bi-directionally sequenced on an ABI 3730XL capillary sequencer (Applied Biosystems) and the resulting traces were edited and assembled into full contigs using CodonCode v. 3.0.1 (CodonCode Corporation).

Because the failure of any of the six fragments to amplify or sequence resulted in missing barcode data (S3 Table), we also sequenced each sample via NGS using the method described in Prosser *et al* [30]. Briefly, instead of amplifying six overlapping amplicons in separate reactions, the same primers were multiplexed in two PCR reactions per sample, and the resulting amplicons from each sample were pooled and sequenced on an Ion Torrent PGM (Thermo Fisher) using a 318 v2 chip and OT2 400bp chemistry (Thermo Fisher). Approximately 4.2M sequence reads were generated, which were demultiplexed into an average of 353K (±143K) reads per specimen. For each specimen, reads were processed to remove primer sequences (Cutadapt v1.8.1) and then filtered based on a minimum quality score of QV20 and a minimum length of 100bp (Sickle v1.33). The reads were then dereplicated (Fastx Toolkit v0.0.14) and assembled into a full-length barcode contig by aligning to a reference barcode sequence [32]. The resulting NGS sequences were merged with their Sanger-based counterparts to fill in the missing data (S4 Table). Final sequences were deposited in GenBank (accessions XXX-XXX) and all NGS reads were deposited in the Sequence Read Archive (accessions XXX-XXX). All of the barcode records are also publicly available in the BOLD dataset “DS-URANIA”, accessed at dx.doi.org/XX.XXXX/DS-URANIA.

### Phylogenetic analysis, statistical parsimony network, and ancestral area reconstruction

Uncorrected Kimura 2 Parameter (K2P) distances for COI barcodes were calculated using modules implemented in BOLD, and subsequently summarized in Excel (Table 1). A Neighbour-Joining tree was also constructed in BOLD under a K2P distance model. Bootstrap values for 1000 replicates were calculated in MEGA 6.06 [33]. To infer a phylogenetic hypothesis for *Urania*, a Likelihood analysis was carried out with PhyML [34] using AIC criterion with bootstrap of 100 replicates and under GTR + *Γ* + *I* + F model as selected by the automatic model-selection module implemented in PhyML. We also conducted a Bayesian analysis in MrBayes v.3.2. [35] under the GTR + *Γ* + *I* model with two simulations, independent Markov chain Montecarlo (MCMC) runs starting from different random trees, each with 3 heated chains and 1 cold chain for 50 million generations.

**Table 1:** Uncorrected p-distances of the mtDNA barcode sequences between *Urania* species.

	*U*. *boisduvalii*	*U*. *fulgens*	*U*. *f*. *poeyi*	*U*. *leilus*	*U*. *l*. *brasiliensis*	*U*. *sloanus*
*Urania boisduvalii* (n = 3)	0.10 ± 0.1					
*Urania fulgens* (n = 15)	2.78 ± 0.1	0.1 ± 0.1				
*Urania fulgens* ssp.*poeyi* (n = 2)	2.53 ± 0.5	0.67 ± 0.4	0.65			
*Urania leilus* (n = 8)	2.54 ± 0.1	2.00 ± 0.1	2.69 ± 0.5	0.04 ± 0.1		
*Urania leilus* ssp. *brasiliensis* (n = 1)	2.55 ± 0.1	1.94 ± 0.1	2.57 ± 0.6	0.02 ± 0.1	-	
*Urania sloanus* (n = 1)	2.39 ± 0.1	1.15 ± 0.1	1.92 ± 0.6	1.44 ± 0.1	1.39	-

Statistical Parsimony Network was constructed in TCS 1.21 with a 90% confidence limit for parsimony [36]. Only full-length (658 bp) COI barcodes were included in the analysis, and with the exception of two *U*. *poeyi* sequences, all those with ambiguous bases were excluded. Dispersal-vicariance optimization of ancestral areas implemented in DIVA 1.1 [37] was used to infer the biogeographic history of the group using five biogeographic units, as follows: SA: South America, CA: Central America, C: Cuba, J: Jamaica, and M) Madagascar. The least number of dispersals (11) was achieved when maxareas was set to 1.

## Results

Male genitalic structure is relatively conserved among *Urania* with the most pronounced differences occurring in the shape and armature of the vesica. We could find no consistent differences in structure between members of two species pairs (*U*. *fulgens* vs. *U*. *poeyi*, *U*. *leilus* vs. *U*. *brasiliensis*), but *U*. *fulgens* and *U*. *leilus* differ in the size and position of diverticula in the vesica, and notably in the absence of the left lateral spine field in *U*. *fulgens*. There are no obvious differences in valve structure between the latter two species, although overall shape may differ subtly. In contrast, *U*. *boisduvalii* differed in vesica structure and possessed a more rounded, elongate valve shape than *U*. *fulgens* and *U*. *leilus*.

**Fig 1:**
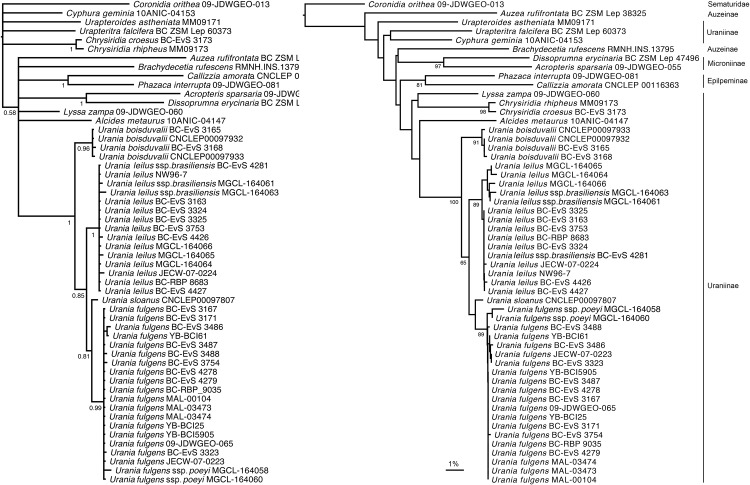
a) Bayesian inference with posterior probabilities, and b) Neighbor-Joining tree with bootstrap support values for Uraniidae COI-5P barcode data.

Our ancient DNA technique [30] produced a full-length DNA barcode (658bp) for *Urania sloanus* and several other species, and fragments of various lengths for other old specimens. Throughout Bayesian, ML (not shown) and NJ analyses, *Urania* formed a highly supported monophyletic group with the same topology, with *U*. *boisduvalii*, *U*. *leilus+ U*. *brasiliensis*, *U*. *sloanus*, and *U*. *fulgens + U*. *poeyi* branching off successively. As expected with mitochondrial data, higher relationships within the subfamily (not of concern in this study) were not supported and the positions of the non-*Urania* taxa varied across our analyses. With *Coronidia* (Sematuridae) used to root the trees, the two representatives of Epipleminae and Microniinae formed monophyletic clades, while Auzeiinae and Uraniinae did not. Within Uraniinae, NJ analysis placed larger species in *Lyssa*, *Chrysiridia*, *Alcides* and *Urania* closer together while the partly nocturnal, possibly non-migratory neighbouring taxa *Urapteritra*, *Urapteroides* and *Cyphura* stayed well outside the remaining genera. These three genera have several other differences from the remaining Uraniinae (smaller size, wing shape, patterning, hind wing venation etc. [11]). Barcode data also could not resolve the sister-group relationship to *Urania*, however *Alcides* appeared to be most similar to *Urania* with no support, while *Chrysiridia* stayed further off in our Bayesian (Fig 1) and ML (not shown) analyses.

Within the monophyletic genus *Urania*, *U*. *sloanus* appeared as sister to but distinct from the Central American species *U*. *fulgens* with moderate support. The three specimens of *brasiliensis* included in our study shared identical haplotypes with *U*. *leilus*, suggesting this taxon is not distinct. Similarly, the two specimens of *U*. *poeyi* were very similar to the Central American *U*. *fulgens*, although they were different at two positions from one another.

## Discussion

The subfamily Uraniinae contains some of the most spectacular Lepidoptera with large and diurnal species whose hindwing tails and contrasting black and iridescent colours often cause them to be mistaken for butterflies. With representatives in the Neotropics, Afrotropics, Oriental and Australian regions, the subfamily shows a typical Gondwanan pan-tropical distribution, corresponding to that of its host plant *Omphalea* [11]. Gondwana began to fragment in the early Jurassic (~180 Mya), and by 110–100 Mya the continents were completely separated [38]. Presuming a vicariance scenario, the split in range of the last common ancestor of *Urania* must have occurred at least 100 Mya in mid- to late Cretaceous, during the final separation of South America and Africa [11]. However, these dates are far before the estimated divergence time for *Chrysirida* and *Urania* (14.1–35.8 Mya) or even the origin of the Uraniidae at 57.3–81.9 Mya [20]. The very low mtDNA differentiation (0.8%) between the two species of *Chrysiridia* in Africa and Madagascar, which were last connected around 165 Mya, also strongly supports one or more recent dispersal events across the Mozambique Channel. The well-documented migratory behaviour and long-distance mass migrations of many species of Uraniinae, including *Urania* [23,39–43] and *Chrysiridia* [44,45] provide an explanation of the more recent dispersal events in these moths.

**Fig 2:**
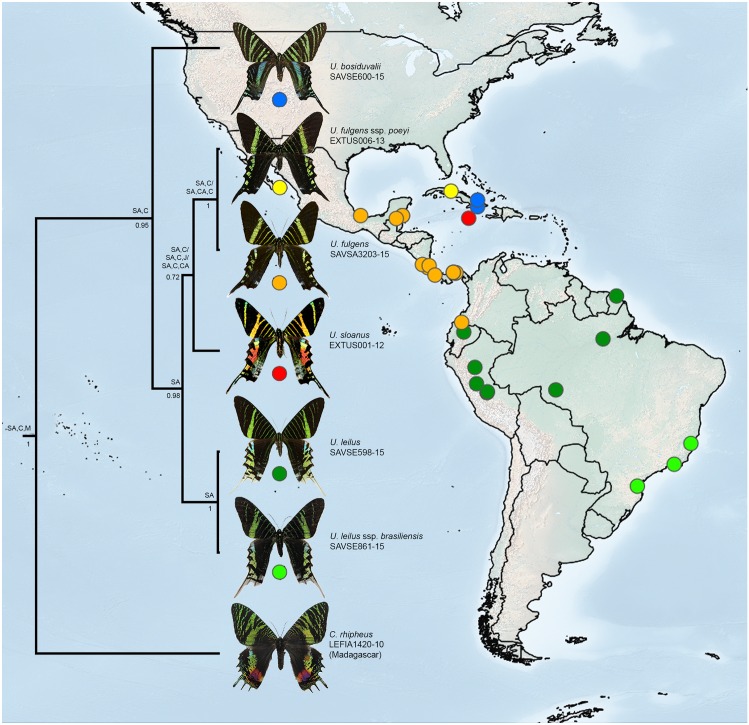
Distribution of examined material and phylogeny of genus *Urania*. Values below nodes are posterior probabilities calculated in BEAST, above nodes are ancestral distributions as predicted by DIVA (SA: South America, CA: Central America, C: Cuba, J: Jamaica, and M) Madagascar).

Dispersal events must also be considered as the likely origin of the Neotropical precursor of *Urania*. The last common ancestor of all *Urania* moths was likely isolated in Cuba and South America in the Early Miocene, where it gave rise to *U*. *boisduvalii* in Cuba (Fig 2). Numerous larval and behavioural autapomorphies have been previously noted for *U*. *boisduvalii* compared to both *U*. *fulgens* and *U*. *leilus* [19]. Identical genitalia and mtDNA sequences support the presence of only one (*U*. *leilus*) rather than two species in South America, however considering the current geographic isolation of these populations, we maintain *brasiliensis* as a subspecies of *U*. *leilus*. The South American lineage dispersed to Central America likely after the formation of the Isthmus of Panama during the Middle Miocene, around 15–11 Mya [46]. The Andes began a rapid uplift in what is now Colombia and Ecuador about 5 Mya, reaching today’s elevations at 2.7 Mya [47]. Genesis of the Andes likely created a natural barrier within the range of ancestral *Urania*, and may have separated ancestral populations of *U*. *leilus* to the southeast of the Andes and *U*. *fulgens* to the west. The taxon *poeyi* seems to be a recent addition to the Cuban fauna as a result of dispersal of ancestral *U*. *fulgens* from Central America, but its geographical isolation from *fulgens* warrants its recognition as a subspecies. The ancestral *U*. *sloanus* also seems to have been isolated in Jamaica following an earlier dispersal of an ancestral lineage of *U*. *fulgens* from Central America. These dispersal events are unlikely to be linked to the geological history of the Caribbean since all *Urania* seem to have diversified relatively recently as evidenced by their low mtDNA divergences (maximum 2.78% between *U*. *bosiduvalii* & *U*. *fulgens*; Table 1), and may be better explained as the result of migratory events aided by the prevailing easterly trade winds [48].

Our molecular analyses do not support the position of the three reportedly nocturnal, non-migratory genera (*Urapteritra*, *Urapteroides* and *Cyphura*) within the Uraniinae, and contrary to Viette’s (1972) interpretations based on wing venation, suggests that *Urapteritra* and *Cyphura* might be sister taxa [49]. Although there is no phylogenetic support from COI-5P for any topology among these three genera, our results seem to hint at a closer affinity between *Urania* and *Alcides* rather than *Chrysiridia* as suggested previously [11,44] and consistent with Heikkilä *et al*’s [22] results based on combined morphological and molecular data. *Urania*, *Alcides* and *Chrysiridia* also share synapomorphies with respect to their tympanic organ [11]. Further evidence is required to clarify a phylogenetic hypothesis between these three genera.

### Taxonomic considerations

The validity of *U*. *sloanus* as a distinct species has never been questioned, and its 1.15% barcode divergence relative to *U*. *fulgens* support its status as a young though valid species. *Urania fulgens* and *U*. *leilus* have previously been treated as being closely related [18] or even conspecific [19]. However, our molecular and morphological studies indicate that they are not sister species, since *U*. *fulgens* is more closely related to *U*. *sloanus* than to *U*. *leilus*. The Andes appear to have been an important dispersal barrier with *U*. *fulgens* occurring on the western slope and north through Central America, while *U*. *leilus* occurs from the east slope and throughout the Amazon Basin, and possibly disjunct on the Atlantic coast of Brazil.

*Urania poeyi* has been treated as a species endemic to Cuba that is questionably distinct from *U*. *fulgens* since the two taxa are very similar in phenotype and larval appearance [11] and share the familial traits of strong sexual dimorphism and migratory behaviour. Their identical male genitalia and lack of mtDNA differentiation further supports their conspecificity. The range of *U*. *fulgens* therefore includes Cuba, either through recent or ongoing dispersal involving populations from Central America, a conclusion supported by the occasional intrusion of this species into Florida and Jamaica [50].

**Fig 3:**
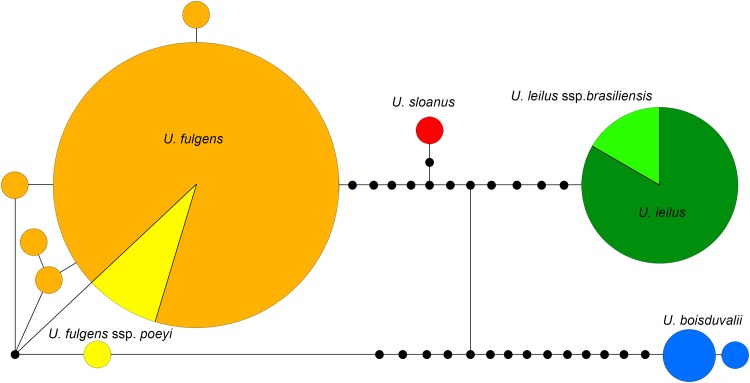
Statistical Parsimony Network of full-length *Urania* sequences examined in this study. Colors correspond to species in Fig 2.

**Fig 4:**
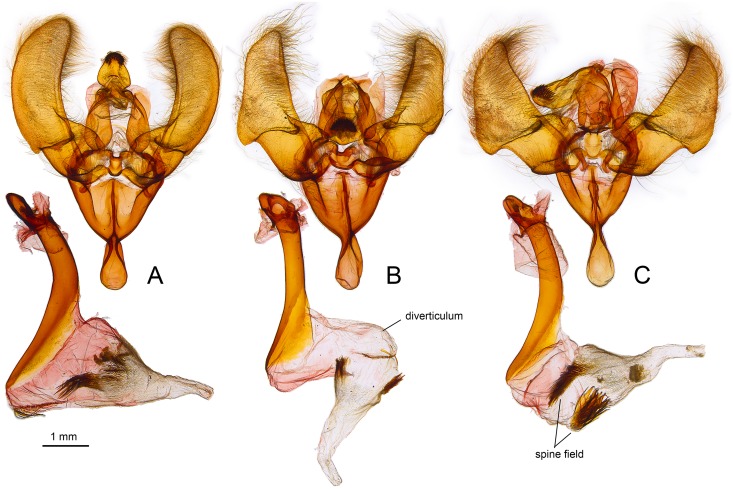
Male genitalia of *Urania* species: A) *Urania boisduvalii* CNCGEO10060, B) *Urania leilus* CNCGEO10062, C) *Urania fulgens* ssp. *poeyi* CNCGEO10061. Genitalia of *Urania leilus* ssp. *brasiliensis* (CNCGEO10059) and *U*. *fulgens* (CNCGEO10058) were identical to B and C respectively and thus not shown.

Similarly, both barcode haplotypes detected in *U*. *brasiliensis* were also found in *U*. *leilus* (Fig 3), and there were no discernible differences in genitalic structures between these taxa (Fig 4). The high degree of similarity in morphology, genitalia and mtDNA between *U*. *brasiliensis* specimens from easternmost Brazil and the Amazonian *U*. *leilus* indicates ongoing or very recent genetic interchange, so we conclude that they are conspecific as suggested by Smith [19].

## Conclusion

Despite the complex and much debated geological history of the Caribbean [51], the evolution of *Urania* in the region seems relatively recent and mostly based on dispersal and subsequent isolation. Island biogeographic theory predicts that most island species are likely to be recent derivatives from mainland populations, and with some exceptions, islands (particularly small islands, here not the case for Cuba) tend to be home not to ancient endemics, but rather to recent offshoots from mainland progenitors. This has important implications for conservation biology, considering that many island populations are threatened. While the loss of island lineages is regrettable, it is important to recognize that closely allied populations often occur on neighbouring mainlands. Understanding the evolutionary relationships between island species and their mainland counterparts is thus a key consideration in island conservation efforts. The two base pairs by which *U*. *fulgens* ssp. *poeyi* is distinct may also flag its conservation significance in Cuba. A similar argument can be made for the anthropogenic islands of the Atlantic forest of Brazil: Only one minute Atlantic forest remnant protects both *U*. *leilus* ssp. *brasiliensis* and its hostplant, *Omphalea brasiliensis* (D. Lees, pers. comm.). Future studies on *Urania* should focus on obtaining full mitochondrial or nuclear genomes for all species, including *U*. *sloanus* but also other Uraniinae, to enable a more comprehensive evolutionary and divergence time analyses.

### Revised classification of *Urania*

***Urania*** Fabricius, 1807

*Urania* Fabricius, 1807; Magazin f. Insektenk. (Illiger) 6: 279. Type-species: *Papilio leilus* Linnaeus, 1758.

*= Urania* [Illiger], 1807; Allgem. Lit. Ztg. Halle [Jena] 1807 (No. 2): 1181 (suppr.). Type-species: *Papilio leilaria* Hübner, [1807].

*= Lars* Hübner, [1807]; Samml. exot. Schmett. 1: pl. [200]. Type-species: *Papilio leilaria* Hübner, [1807].

*= Cydrus* Billberg, 1820; Enum. Ins. Mus. Billb.: 81. Type-species: *Papilio leilus* Linnaeus, 1758.

*= Cydimon* Dalman, 1825; K. VetenskAcad. Handl. 1824 (2): 407. Unnecessary replacement name for *Urania* Fabricius, 1807.

*= Leilus* Swainson, 1833; Zool. Illustr. (2): pls. 125–126, 129–130. Unnecessary replacement name for *Urania* Fabricius, 1807.

*= Dasycephalus* Walker, 1854; List Spec. Lepid. Insects Colln Br. Mus. 1: 4. Unavailable name.

*= Uranidia* Westwood, 1879; Trans. Zool. Soc. Lond. 10: 516, 520–52. Unnecessary replacement name for *Urania* Fabricius, 1807.

*= Lelius*; Swainson, 1836, Nat. Hist. Classif. Birds 1: 233. Misspelling.

**1. *Urania boisduvalii* Guérin-Meneville, 1829**

*Urania boisduvalii* Guérin, 1829, Icon. Ins., p. 490. Type locality: Cuba. Type lost.

*= Urania fernandinae* MacLeay, 1837; Trans. Zool. Soc. Lond. 1: 180. Type locality: Cuba.

**2. *Urania fulgens* Walker, 1854**

*Urania fulgens* Walker, 1854, List Lep. Het. Br. Mus. 1, p. 5. Type locality: “Sta. Fe de Bogota”, “Mexico”. Syntypes in BMNH. Walker (1854) lists two specimens “from Mr. Argent’s collection”; Mexican specimen missing (A. Zilli, pers. comm.).

*= Cydimon cacica* Guenée, 1857, Spec. génér. Lep. 9(9): 8. Type locality: “Mexique: Environs d’Acapulco”.

**2a. *Urania fulgens poeyi* (Herrich-Schäffer, 1866) revised status**

*Cydimon poeyi* Herrich-Schäffer, 1866, Corr. Blatt. Ver. Regensb. 20, p. 135. Type locality: Cuba. Type not located; possibly in Gundlach collection, Havana (currently inaccessible).

**3. *Urania leilus* (Linnaeus, 1758)**

*Papilio leilus* Linnaeus, 1758, Syst. Nat. 10: 462. Type locality: America. Type in Uppsala University Zoological Museum, Sweden.

*= Papilio leilaria* Hübner, [1807], Verz. Bek. Schmett.: 289. Unjustified emendation of *Papilio leilus* Linnaeus, 1758.

*= “leilus” surinamensis* Swainson, 1833, Zool. Illustr. (2)3: pl. 125. Type locality: [Surinam].

*= Cydimon amphiclus* Guenée, 1857, Spec. génér. Lep. 9(9): 7. Type locality: Trinidad.

*= Urania curvata* Pfeiffer, 1917, Ent. Z. 31: 70. Type locality: “Puerto Patinm [sic] in Bolivien”.

*= Urania leilus* ab. *elegans* Niepelt, 1930, Ent. Z. 44: 18. Type locality: Bolivia.

*= Urania leilus* var. *intermedia* Pfeiffer, 1917 (in litt.).

**3a. *Urania leilus brasiliensis* (Swainson, 1833) revised status**

*Leilus brasiliensis* Swainson, 1833, Zool. Ill. (2)3, pl. 126. Type locality: “Brazil”. Type in BMNH.

**4. *Urania sloanus* (Cramer, 1776)**

*Papilio sloanus* Cramer, 1776, Pap. Exot. p.134. Type locality: “Jamaika”. Type in BMNH.

*= Leilus occidentalis* Swainson, 1833, Zool. Ill. 2(3), pl. 129. Type locality: Jamaica.

*= Urania sloanaria* Hübner, 1816, Verz. Bek. Schmett.: 289. Type locality: Jamaica. Unjustified emendation of *Papilio sloanus* Cramer, 1779.

*= Urania sloaneus* Herbst (misspelling).

## Supporting Information

S1 Table Material examined, localities and GenBank accessions. (XLS)

S2 Table List of non-tailed primers used in Sanger sequencing to amplify six short, overlapping fragments of the COI-5P barcode region. (XLSX)

S3 Table Detail on the technologies used—Sanger or NGS—to successfully amplify each fragments (F1-F6) of COI-5P barcode region. To see additional information on primer combinations, reactions attempted on each sample, and download the trace files please go to http://dx.doi.org/10.5883/DS-URANIA.
(XLSX)

S4 Table Min, max, and average base coverage for 10 samples amplified using NGS post-QC read filtering. Although the minimum coverage was 0 for all samples (because none of the samples had 100% success), for the final consensus sequence we generally only included regions with a minimum coverage of 10, unless we were absolutely certain that the low-coverage reads were accurate.
(XLSX)
